# The Medicine of the Future

**Published:** 1892-09-24

**Authors:** 


### The Hospital

AN INSTITUTIONAL JOURNAL OF

Science, Medicine, Nursing, and Philanthropy.

*For Hospitals, Asylums, Infirmaries, Dispensaries, Medical Practitioners, Students; Nurses, and the Charitable Public.*

VOL. XII.—No. 313.] [SEPT. 24, 1892.

### THE MEDICINE OF THE FUTURE.

MR. GLADSTONE, as the *Pall Mall Gazette* informs the public, entertains what until recently would have been considered novel opinions as to the degree of culture already attained by the medical profession, and as to the position its members are likely to occupy in the front ranks of thought and leadership in the near future. It seems to have been stated in a German professional work that Mr. Gladstone had prophesied that doctors were destined to become the leaders of the people. The usual "correspondent" took up the matter and wrote to the veteran statesman for confirmation or contradiction. Mr. Gladstone, always courteous, wrote in reply: "So far as regards the exact words cited in your letter I cannot positively say aye or no, and I rather think that I should in using them have added some qualifying or limiting expressions. But it is certainly a fact that for a very long time I have believed the medical profession to be both in a state of absolute advance from the progress of its science—this it may be said is a mere commonplace—and of relative advance from the particular features attaching to our civilisation in its onward movement."

It is peculiarly fitting that young men—and shall we not add, young women—who are just about to commence their first year of professional study, with a view to ultimate graduation in medicine, should have their minds directed to the comparative and public aspects of the medical profession so prominently brought into relief by Mr. Gladstone's judicial estimate. The idea that doctors should be in a wide political and social sense leaders of the people is decidedly novel. But is the prophesy likely to be fulfilled? Is it indeed already in course of fulfilment? Is it the case that doctors know many things which other educated people do not know; and are those things which the doctors know the germinal factors which will influence and to a large extent make the social and intellectual life of the future? Cultured doctors undoubtedly not only know more of certain departments of science and thought than other classes of educated men, but they know so very much more that they may be said to live to a large extent in another world, apart by themselves. There is a whole hemisphere of science and literature of which the educated world which is out side medicine has hardly yet learned so much as the language; in many cases not even the alphabet.

But are the science, the literature, and the thought which constitute the staple of the doctors' mental belongings destined to displace and supplant the older mental forms and properties which have constituted the chief intellectual wealth of past generations? It seems certain that they are to a very considerable extent. Take literature! Is it alive, or is it dead? The novel is alive, but what of the poem, and the play? What, too, of philosophy? Never were there so many men devoted to writing; never was there so little literature of a kind which is destined to survive even the life of a single generation! Is it not the truth that all men of any considerable intellectual power who find themselves in middle age bound to pure literature as a means of livelihood, are so sick and weary of the business that nothing but sheer necessity compels them to go on. Pure literature, for the present at any rate, is exhausted. Writers are in the position of the prisoners in the Black Hole of Calcutta; they are breathing the literary atmosphere breathed so many times before by past generations that they can hardly get enough pure oxygen out of it to keep their souls alive.

But now turn, on the other hand, to medicine—to medicine and the sciences associated therewith. They are throbbing with the intensest life. That young men in scientific laboratories, who have name and fame yet to make, should be full of kindling enthusiasm is not to be wondered at. But here is the surprising thing that middle-aged and old men are as eager, and even more eager, if that be possible, than the younger. What an astonishing sight it was a year or two ago to see half the medical world flock to Berlin in midwinter in order to verify for themselves the results of a single method of treatment in a single department of medicine. It is true they were bitterly disappointed. But the very same thing would happen again to-day if a trusted man of medical or other science were to announce such a discovery as Koch announced.

Men have laboured with infinite pains to understand mind and its life in the past; now they are turning, or have turned, to the body. It is obvious that even the students of mind themselves have felt compelled to turn to the body, because they have at length realised that mind itself can by no means be fully understood except as it is approached through the body. These are common-places to the cultured medical man: they are questions for fierce wrangling and disputing to those who know only the ancient literary culture. But whilst the angry disputants of the old order are contending over mere names and definitions in a state of semi darkness, the men of science and scientific literature have galloped off ahead and are out of sight. The facts, the actual substance of the sciences with which medical men deal, are of the primest importance to the great world, and will be discovered to be so in no long time. They constitute the new America and Australia to which even literary and religious men will ultimately be driven by the sheer stress and straitness of old ideas and institutions. To the literary man they offer new fields of thought; to the theologian new revelations.

But if the substance of the medical and allied sciences be of vital importance to the world generally, the methods of the laboratory and of the clinique are more transcendantly important still. Here, in the simplest language, is the difference between the method of the old culture and that of the new! The old says, "It must be so, because, etc."; the new says, "I cannot tell whether it must be so or not; but here are the actual facts, and I will examine them and see if it is so." The method of fresh investigation is the new method; and it will soon be the only possible one. All men see its fitness in medicine and kindred sciences; but it is equally fit for all departments of activity, and it must of necessity be adopted. The statesman must employ it whether he will or not, and so, too, must the theologian, and the student of social dynamics and statics. The *a priori* has had its day; the inductive must now come in. Physicians, who are "old hands" at the inductive, must take precedence of those who are yet in their philosophic childhood: they will be leaders by the sheer force of the inevitable. It is wonderful how slow the world is to learn the importance of what it calls "mere method." Here is the importance: The native African still fights with a club or a spear; the European fights with a rifle. This difference of mere method makes the European at least twenty times more effective than the native African.

No modern young man ever yet regretted having had a medical education. Many a one may have regretted the choice of medicine as a profession; nay, many a one finds it so crowded and ill paid, that he does regret it. But the methods, the thoroughness, the absolute honesty and sincerity of everything connected with modern medical education are beyond all price. A young man with money and leisure should study medicine by all means, whether he ever intends to practice it or not. Its substance and its methods, but especially its methods, constitute the most liberal of all educations. He who has once thoroughly learnt the methods of medicine will never practice any other; and practising these, in whatever department of human activity his lot may be cast, he cannot fail to be a man.

